# Human–robot collaboration in surgery at the nexus of knowledge, agency, and ownership

**DOI:** 10.1038/s41598-025-08437-w

**Published:** 2025-07-02

**Authors:** Ezgi Merdin-Uygur, Selcen Ozturkcan, Mustafa F Özbilgin, Faruk Yılmaz, Özgür İnce

**Affiliations:** 1 Brunel Business School, Brunel University of London, Uxbridge, UK; 2 School of Business and Economics, Linnaeus University, Växjö, Sweden; 3 Sabanci Business School, Sabanci University, Tuzla, Turkey; 4 Muş Alparslan University, Muş, Turkey; 5 Inclusens GmbH, Kulmbach, Germany

**Keywords:** Robotic surgery, Healthcare robots, Human-robot relationship, Knowledge, Agency, Ownership, Professional ignorance, Psychology, Health care, Health occupations, Engineering

## Abstract

Human-robot collaboration is transforming healthcare, particularly in surgical environments. Robotic surgery systems, embodied by advanced AI, are pivotal in augmenting human expertise across specialties such as gynecology and laparoscopic surgery. However, critical gaps remain in understanding how knowledge, agency, and ownership shape these collaborations. We address these gaps through semi-structured interviews with eleven healthcare professionals from diverse surgical roles. Our findings reveal that while robotic systems enhance precision and efficiency, they also generate tensions related to professional autonomy, control, and responsibility. Participants expressed ambivalent views, simultaneously demonstrating trust in the technology and strategic disengagement to preserve human authority. Concepts such as *avatarization*, the perception of robots as extensions of the self, and *strategic ignorance* emerged as key mechanisms through which professionals manage this evolving relationship. These dynamics point to the need for rethinking human–robot roles as fluid and co-constructed rather than fixed or hierarchical. We also emphasize the possible use of robotic systems to promote inclusivity and accessibility in healthcare while identifying structural barriers such as high costs, dependence on proprietary technology, and uneven organizational readiness. Our research enhances theoretical frameworks on human–robot interaction, providing practical and conceptual insights for the creation of equitable, sustainable, and context-sensitive robotic healthcare systems.

## Introduction

Human-robot collaboration is transforming healthcare, particularly in surgical settings where robotic systems, such as the Da Vinci Robotic Surgery System, enhance precision and efficiency in surgeries^1^. As a “Level 1” system on the Levels of Autonomy in Surgical Robots (LASR) scale, the Da Vinci Robotic Surgery System provides minor adjustments, such as tremor correction, while requiring full human control^2^. Despite their potential to improve patient outcomes and expand access to care, these systems introduce complex dynamics around knowledge, agency, and ownership, challenging traditional roles and power structures in the operating room^3^.

While robotic surgery offers benefits like reduced recovery times, it also raises concerns about professional autonomy, team dynamics, and economic barriers. Studies indicate that human-robot teams may underperform compared to humans or robots alone due to misaligned expectations and dependencies^4^. Recent evidence also highlights increased bile duct injuries in robotic-assisted procedures, suggesting potential over-trust in human-robot integration^5,6^. These challenges necessitate a critical examination of how healthcare professionals adapt to robotic systems, particularly in high-stakes surgical environments.

Drawing on multidisciplinary perspectives from psychology, sociology, and public policy, this study introduces conceptualizations novel to this domain (i.e., avatarization and strategic ignorance) to explore how knowledge, agency, and ownership shape human-robot collaboration in surgery. Avatarization describes the perception of robots as extensions of human expertise, while strategic ignorance reflects professionals’ selective disengagement to preserve autonomy. Through semi-structured interviews with eleven healthcare professionals in a Turkish public hospital, this research aims to investigate how these dynamics influence interactions with robotic systems, offering insights for equitable and sustainable integration in healthcare^7^ and beyond^8–10^.

## Theoretical background

### Professional ignorance and hierarchies of knowledge, agency, and ownership between humans and robots in healthcare

This paper investigates three historically entrenched relationships: knowledge, agency, and ownership, as their fundamental assumption of human superiority requires reevaluation by rapid technological advances in healthcare robotics. Human-robot collaboration involves complex dynamics shaped by the unpredictable and often unstructured nature unique to healthcare settings. This framework stands in contrast to the more controlled environments, such as manufacturing, where robotic systems have traditionally operated^11^. In clinical practice, robots are deployed across a range of functions: Paro and Justocat provide companionship in elder care, TUG handles logistical tasks, and the Da Vinci Robotic Surgery System supports surgical procedures that demand high precision and control^12,13^. As a Level 1 system on the LASR scale^2^, it requires continuous human operation, integrating substantial physical and cognitive engagement from its users^14^. As robotic platforms expand surgical precision, they simultaneously reshape task distribution and spatial configurations in the operating room^3^, reflecting more profound sociological issues surrounding knowledge, agency, and ownership.

In the knowledge domain, healthcare professionals often prioritize “sufficient knowledge” without mastering every detail in their field^15^. Yet, literature has primarily addressed patient ignorance regarding genetics^16^ and mental health^17^, while the Da Vinci Robotic Surgery System supports surgeons by automating certain tasks but still requires human intervention and oversight^2,18^. Yet, human professionals sometimes experience a diminished sense of control, even when acting as primary operators. Terry^19^ warns that viewing robots solely as tools fails to capture the complex interactions occurring in medical environments, where robots may act with varying degrees of autonomy and influence. For example, Level 2 or higher on the LASR scale raises important questions about shared responsibility and the distribution of control^2,20^. When performing robotic surgeries remotely, for instance, surgeons depend on team members for feedback and assistance in executing commands, highlighting a shift in power dynamics within the surgical team^21^.

The third axis, ownership, reflects evolving dynamics in the relationship between humans and robotic systems in healthcare. These technologies are typically owned by hospitals or external technology firms, and the economic value they generate tends to accrue to those institutional owners rather than to the professionals who operate them. This asymmetry reinforces existing income inequalities, as robotic contributions, unlike human labor, remain untaxed and unshared with those directly responsible for their use^22^. Simultaneously, a form of psychological ownership develops among surgeons and clinical staff, who engage closely with these systems and feel that an object or system is “theirs,” observed in hands-on practices such as robotic surgery^23^. This ownership is shaped by factors such as the surgeon’s control over the robot, the time and effort invested in mastering the technology, and familiarity with the system through repeated use^24,25^. However, robotic surgery involves not only the surgeon but also a team of healthcare professionals who contribute to the system’s operation. This broader involvement underscores the necessity of shared ownership, where nurses, technicians, and other supporting staff share responsibility for the robotic system’s successful integration and performance^26^.

These conceptual dimensions of knowledge, agency, and ownership served as the analytical lens through which we examined the interview data. In the following section, we present the results structured around these themes, beginning with how healthcare professionals define and interpret the Da Vinci Robotic Surgery System through their language and acts of strategic (dis)engagement.

## Results

### Words that mold knowledge and strategic ignorance

What defines the relationship between robots and humans is what humans know or ignore about robots and human acts of knowing and ignoring robots. Between knowledge and ignorance, defining is the most significant act through which humans frame their relationship with robots. Acknowledging the critical role of definitions in shaping how healthcare professionals perceive and describe robotic surgery systems, we first highlighted the lexical choices and descriptive words that healthcare providers employ to define and classify robotic surgery systems, showcasing the shaping of anthropomorphism within the spectrum from machines to autonomous entities. The definitions of surgery robots provided by the interviewees ranged from “an assisting tool” (Fatma, a female, ear, nose, and throat surgeon with 5 to 10 years of robotic experience) to “technology” (Kaan, a male cardiovascular surgeon, associate professor with 5 to 10 years of robotic experience), and “technological material” (Ali, a male, urology surgeon, professor and education manager with 5 to 10 years of robotic experience).

We highlight important glimpses from the interviews that showcase the strategic dynamics of knowing and ignoring the robots at the same time:

Robots are a type of tool and belong to the class of tools. The robot doesn’t perform the surgery itself. Just like you open and close the tip of the scissors from the holding point while cutting, the same logic applies to the robot. In the robot, the surgeon opens and closes a device like a joystick. Like the tip of the scissors, the arms of the device open and close inside the patient. They are things produced by human knowledge and effort. It’s inappropriate to compare them with humans; they are the servants of humans (Ada, a female nurse with 5 to 10 years of robotic experience).

Not only surgeons but also various healthcare professionals, such as nurses, emphasize the role of robots as tools akin to scissors, underscoring the human surgeon’s control by consistently stating that robots are tools, not competitors. They highlight the role of robots in enhancing human abilities and efficiency, dismissing the notion of competition with humans. They draw a comparison between human surgeons using robots and excavator operators using tools, highlighting their functional equivalence in enhancing efficiency.

There is no purpose in competing with the robot; they are not on the same level. The one who makes, uses, and discards the robot is human. Ultimately, what matters is the robot serving humans. Previously, people used to dig manually with a pick and an axe. Now they do it with a tractor or a machine. If you replace the driver with a robot, it remains a tool. In short, robots are a type of tool and belong to the class of tools. They are things produced by human knowledge and effort. It’s inappropriate to compare robots with humans; they are the servants of humans. If you use these tools effectively, work efficiency increases, and human welfare increases. Using the term ‘robotic surgeon’ in the health field seems ridiculous to me. You are the one using the robot; you are the worker. The logic behind a surgeon operating the Da Vinci system is similar to that of an excavator operator using their equipment. In this sense, what surgeons do is equivalent to what excavators do (Ali, a male urology surgeon, professor and education manager with 5 to 10 years of robotic experience).

Acts of knowing and ignoring shape the limits of how humans define their relationships with robots. Consequently, defining becomes a political act, setting the parameters of human-robot interactions.

### Agency: dual skeptic projections

Interviewees sometimes referred to their knowledge bases of popular notions, images from popular culture, or terms related to cultural references, technology trends, and future visions, based on their lexical choices in constructing the meaning of robots to know and ignore them. Signaling the key theme of possessing agency that differentiates tools from humans, the interviewees` projections as to how human-robot collaboration will reach the future and the future of healthcare consisted of two distinct themes touched upon without prompt. These were either fully autonomous AI-powered healthcare service providers (i.e., robot doctors or surgeons) or support personnel at the other end of the autonomy continuum (i.e., pill distribution or secretarial robots). For example, there were references to `waiter,’ which is a swiftly growing and highly visible role for robots. Yet, they highlight the disparity between popular perceptions of AI robots and the reality of surgical robotics like the Da Vinci Robotic Surgery System, prompting speculation about future developments:

The robot we currently use is not actually the artificial intelligence robot that comes to mind classically for people. Of course, nowadays, there are waiter robots providing service, but the Da Vinci robot we use is not that type of robot. Undoubtedly, the future will reveal the development of such robots and their potential applications in medicine. This perception of robots differs from the actual capabilities of the Da Vinci robot (Can, a male urology surgeon and manager with 5 to 10 years of robotic experience).

Without any prompt and regardless of their roles, the interviewees referred to AI technologies and therefore fully embodied AI, also known as robots, such as humanoid, entirely autonomous surgeons. However, almost all interviewees declared this vision highly improbable in the foreseeable future. While some referred to the future of human capital and the healthcare provider’s human job market, others mentioned ethical concerns as well as domains of control between humans and technology.

Routine healthcare robot future scenarios signaled that interviewees were overly skeptical. They anticipated that human-AI integration in surgery would strengthen decision-making abilities, but it could also reduce reliance on healthcare professionals and negatively impact employment.

With the advancement of technology in healthcare services, human-robot interaction will increase in patient care and other service processes. I am uncertain whether this advancement will ultimately benefit humanity. Previously, before the machines for everything came out, people used to do more activities. The limitation of human activities by these machines and robots brings up increasing health problems. Examples include chronic fatigue and obesity (Sarp, a male general surgeon with less than 5 years of robotic experience).

### Strategic rewriting between knowledge and agency: equity, inclusivity, and sustainability

Despite the skepticism over the future of human-robot partnerships, references were made to the equalizing and inclusive potential of such collaborations to close the access gaps for restricted groups or populations. Some surgeons refer back to the origins of robotic surgery systems, such as the NASA plan’s main motivation to be able to perform surgery on astronauts in space from the Earth to set the stage for the future dream of humanity. This origin story set the context and laid the background.

The idea emerged to operate on NASA astronauts from Earth or for remote surgeries. The use, such as operating on a patient in one country from another, was requested but not well received ethically. Perhaps in the future, developments enabling a surgeon to operate on a patient remotely may occur in a shorter period of time (Can, a male urology surgeon and administrative manager with 5 to 10 years of robotic experience).

The rest of the references to equality, accessibility, and inclusion had the common view of fostering collaboration between humans and robots to bridge gaps, ensuring equal opportunities and access in various spheres of life.

When standardization is achieved, inequalities, such as receiving service from the best surgeon, can be eliminated (Mesut, a male urology surgeon assistant with less than 5 years of robotic experience).

These concluding remarks paint a future portrait of a human-robot partnership for all, striving for harmonious coexistence and collaboration between humans and robots, emphasizing inclusivity and shared benefits for everyone involved.

### Between agency and ownership: avatarization, not automation

Our analysis revealed various forms of agency that humans and robots manifest in their relationship, expanding and curtailing each other’s possibilities of decision-making and action. The most striking example amongst them was how most interviewees visualized an amalgamation between the human surgeon and the robotic surgery system. As there is an advanced degree of synchronization between the surgeon’s hand movement and the robotic arms` movements in the operating room, the surgeon perceives the robot not as a team member but rather as their avatar touching, seeing, and moving within the patient. The movement, capability, and success of the operation are directly attributed to the human surgeon, while failures are also owned by the human surgeon similarly.

Providing the robot with three-dimensional vision gives the feeling that you have entered the patient (Ece, a female anesthesiologist with 5 to 10 years of robotic experience).

The transformation of a robotic surgical system into an extension of the surgeon’s body and capabilities creates the perception of a seamless union of human expertise and robotic precision through a merging of their agentic possibilities. The avatarization image also encapsulates sensory synchronization, meaning achieving synchronization between the surgeon’s senses and the robot’s feedback loops. The perceptual integration of the human surgeon’s expertise and consciousness with the robotic surgical system leads to a sense of oneness and, therefore, a shared agency over success and, surprisingly, over failure.

The robot surgeon does whatever command is given. Any mistake here would be the surgeon’s fault. […] It operates based on the commands given by the surgeon. Therefore, there is no error or deficiency because the surgeon is directing (Kaan, a male cardiovascular surgeon with 5 to 10 years of robotic experience).

In conclusion, humans allow or deny possibilities of agency for themselves and robots. In the case of fusion, humans give significant agency to themselves and robots in their engagement. Nevertheless, the avatarization frames human agency as superior to robots, who are again instrumentalized in the relationship. Interestingly, the fusion and avatarization perceptions seemed to be put aside when discussing the critical issues of ownership, such as power. While avatarization reflects a sense of control and ownership over robotic systems, it may also foster over-trust in one’s abilities. This perception of seamless fusion between human and machine can obscure risks and lead to complacency. Recent findings on increased bile duct injuries in robotic-assisted surgery highlight such concerns^5,6^. Although our participants emphasized human responsibility, the belief that “the robot does whatever command is given” may reinforce a false sense of infallibility. As robotic autonomy evolves, addressing this potential overconfidence through training and reflective practice is essential.

### The ownership games

Most interviewees declared human-centric ownership, emphasizing the surgeon’s pivotal role in owning, controlling, and overseeing the process for the future as well as for the present. Distinguishing between the surgeon’s intrinsic control in surgical decision-making and the dependency on external robotic tools also implies a nuanced power dynamic of ownership structure:

Maybe even the robot can perform surgery on its own with the commands given by the surgeon in advance. In such a case, there may not even be a need for a surgeon. Of course, such a development would not be a good thing for surgeons. Like the Industrial Revolution, which reduced the need for human labor with machines, the integration of robots with artificial intelligence may have a similar effect (Sarp, a male general surgeon, associate professor with less than 5 years of robotic experience).

Yet again, there were dualities in future projections, where robots were allowed and even encouraged to take ownership.

I believe that humanity will develop technology in such a way that it will dominate (Emin, a male neurosurgeon, associate professor, and deputy chief, with no robotic experience due to his managerial role).

### Between ownership and knowledge: comprehensive stakeholder perspectives

Throughout the planning and execution of this research, understanding the perspective of the whole service team has been crucial, given the necessity for a collective understanding and competency among the surgical team beyond just the surgeon in effectively utilizing robotic technology during a surgical procedure. The necessity of collaborative proficiency in robotic surgery acknowledges the surgeon-robot’s roles (i.e., providing direct warnings and enhancing precision during surgery by alerting the surgical team), the nurse team’s roles (i.e., docking and preparing proper covers on the robot’s arms), and administrative roles, stressing the importance of preparedness and adaptability within the whole organization.

… on the day of the surgery, it’s not only the surgeon who will use that robot on the operating table, but everyone else besides the surgeon must also be competent in this matter (Kaan, a male cardiovascular surgeon and associate professor with 5 to 10 years of robotic experience).

The cases provided by the interviewees aligned with the literature’s assertion that robot-assisted techniques lead to improved patient outcomes and compensate for their limitations, such as the lack of physical touch. Many noted the impracticality of solely relying on robotic surgery due to its high costs. While the initial costs of robot-assisted surgery are higher, they may be offset by long-term savings from reduced complications and revision surgeries. Thus, interviewees underscored both the practical advantages and the economic considerations of integrating robotic assistance in surgical procedures:

A while ago, we were applying robotics to a wider spectrum of cases, such as cardiovascular, general surgery, urology, ear, nose, throat, gynecology, and robotic transplantation. However, due to the increase in oncology patients waiting to receive service, the Ministry of Health was asked to accept/prioritize only oncology patients. Following this, restrictions were imposed on applications due to the increase in costs… I can say that, especially purchasing the material with the exchange rate, makes the use of the robot very difficult at this point… When these parts expire, they cannot be reused and are destroyed. For this reason, the company being decisive in these decisions is an important weakness, as it increases technology dependency (Emel, a female nurse with 5 to 10 years of robotic experience).

## Discussion

This study explored the dynamics of knowledge, agency, and ownership in human-robot collaboration within surgical settings, offering new perspectives on how professionals adapt to and resist the integration of robotics in high-stakes environments. By analyzing healthcare professionals’ experiences, this research introduces novel insights that extend existing theories on human-robot interaction while challenging common assumptions about the benefits and impacts of automation.

A significant contribution of this research is the introduction of two novel concepts, i.e., avatarization and strategic ignorance, which enhance our understanding of human-robot collaboration. Avatarization captures how robots are perceived as personalized extensions of human expertise, where robotic systems feel like seamless tools of professional intent. For instance, surgeons often described their experience as if the robot were “one with them,” reflecting the fusion of human expertise and robotic precision^27^. This perspective moves beyond existing frameworks that view robots as static tools or teammates and emphasizes the dynamic interplay between human intent and technological capabilities.

Strategic ignorance, on the other hand, reveals how healthcare professionals deliberately disengage from robotic capabilities to maintain control, autonomy, and professional identity. This selective engagement underscores a tension between the empowerment offered by robotic systems and the desire to preserve traditional roles. Such behaviors highlight how humans balance their reliance on technology with the need to assert their agency. These findings will be increasingly relevant when the next levels of autonomy are investigated (Fosch-Villaronga, 2023), since the increased autonomy level could affect the surgical human-robot collaboration in new ways and create a shift in the power dynamics^5^; Mullens et al.^6^.

In the literature on automation, robots are commonly framed as tools, teammates, or leaders^28^. The interviewees in this study consistently described surgical robots as tools that enhance surgeon control and efficiency, with little indication of perceiving robots as competitive entities. This aligns with the “automation as tool” paradigm, where robots are considered subordinates to human “masters”^28^. However, our findings go further, revealing how healthcare professionals reshape their relationship with robots, viewing them as co-creators of expertise rather than mere subordinates.

Our findings reveal critical tensions in the dynamics of agency and ownership. While robotic systems empower healthcare professionals^29^ by enhancing precision and reducing physical strain, they also introduce dependencies that challenge professional autonomy. For example, surgeons often reported a strong sense of psychological ownership over robots, but this sense of ownership contrasts with the institutional and corporate control that ultimately governs the use of these systems. This duality creates a paradox, where professionals feel empowered yet constrained by broader organizational structures.

The redistribution of agency within surgical teams further illustrates these tensions. Nurses and anesthesiologists described how their roles often became subservient to the robot’s requirements, reducing their agency and altering team dynamics. This reallocation of responsibility underscores how automation can disrupt existing hierarchies, sometimes leading to perceptions of diminished roles among non-surgical team members.

The literature on AI futures generally spans a broad spectrum^30^. At one end, AI is celebrated as a transformative revolution with lasting impacts; at the other, concerns are raised about machines surpassing human capabilities. For instance, being replaced by robots is often associated with a larger perceived threat to one’s economic future^31^. Between these extremes, moderate projections suggest a gradual integration of automation, allowing ample room for policy, ethical, and workforce adaptations^32^. Our findings situate themselves within this discourse, showing how healthcare professionals navigate these tensions through behaviors like strategic ignorance and selective engagement, which mediate the broader impacts of automation.

Among the themes related to knowledge and agency, a prominent focus in this study is the potential for robotic systems to create accessible, sustainable, and inclusive futures. Tracing the history of surgical robots reveals their role as solutions to healthcare disparities. For example, NASA’s initial vision of using robots for remote surgery on astronauts exemplifies how such technologies can transcend geographical barriers to improve healthcare access^33^. Interviewees in this study envisioned similar futures, where human-robot teams collaborate to overcome limitations and ensure inclusivity in healthcare delivery.

However, these optimistic visions are tempered by practical concerns. The high operational costs of robotic systems and their reliance on proprietary parts present significant sustainability challenges, particularly for resource-constrained healthcare systems. These issues exacerbate existing disparities in access to advanced healthcare technologies and raise important questions about equity. Policymakers must address these barriers to ensure that the benefits of robotics are distributed fairly across healthcare systems, prioritizing equitable access and training.

The healthcare sector, as a fundamental pillar of society, has an important effect on human interaction and well-being across all stages of life^34^. This study highlights how robotic systems, if implemented thoughtfully, can enhance this role by improving precision, reducing recovery times, and expanding access to care. However, achieving these outcomes requires addressing the tensions and contradictions outlined here, ensuring that the adoption of robotics aligns with broader goals of inclusivity and sustainability. We acknowledge that the study’s limited sample size, drawn from a single public hospital, constrains the generalizability of findings and calls for caution in applying them across diverse contexts.

## Methods

### The research context

This study examines the use of the Da Vinci Robotic Surgery System among healthcare professionals. Initially approved by the U.S. Food and Drug Administration (FDA) in 1997, the system was originally limited to visualization and tissue retraction tasks^1^. The Da Vinci Robotic Surgery System investigated in this study is a “Level 1” on the autonomy scale LASR^2^ that delivers only minor adjustments to the surgeons’ hand movements (e.g., tremor correction), with the decision-making relying on the surgeon. Still, our findings from this level of autonomy in the robotic surgery systems are also increasingly relevant, especially when the higher levels of autonomy are investigated, since such could affect the surgical human-robot collaboration in new ways and create a shift in the power dynamics. The Da Vinci Robotic Surgery System also facilitates telemedicine by enabling surgeons to operate on patients remotely, removing the need for physical presence in the operating room.

The healthcare industry is highly regulated worldwide; however, regulatory frameworks for qualitative research involving healthcare professionals vary significantly across national contexts. In many countries, such as Sweden, the UK, and the US, even non-interventional studies with medical personnel often require strict board approvals. While ensuring ethical rigor, such requirements may limit early-stage, exploratory research such as ours. In contrast, Turkey’s regulatory framework allows greater flexibility for qualitative studies involving healthcare professionals. In this context, we obtained approval from a local university ethics committee and conducted the study in full accordance with established ethical principles, including voluntary informed consent and confidentiality. This policy environment enabled broader access to multidisciplinary surgical staff of varied specialties and shaped both our site selection and research design.

**Fig. 1:**
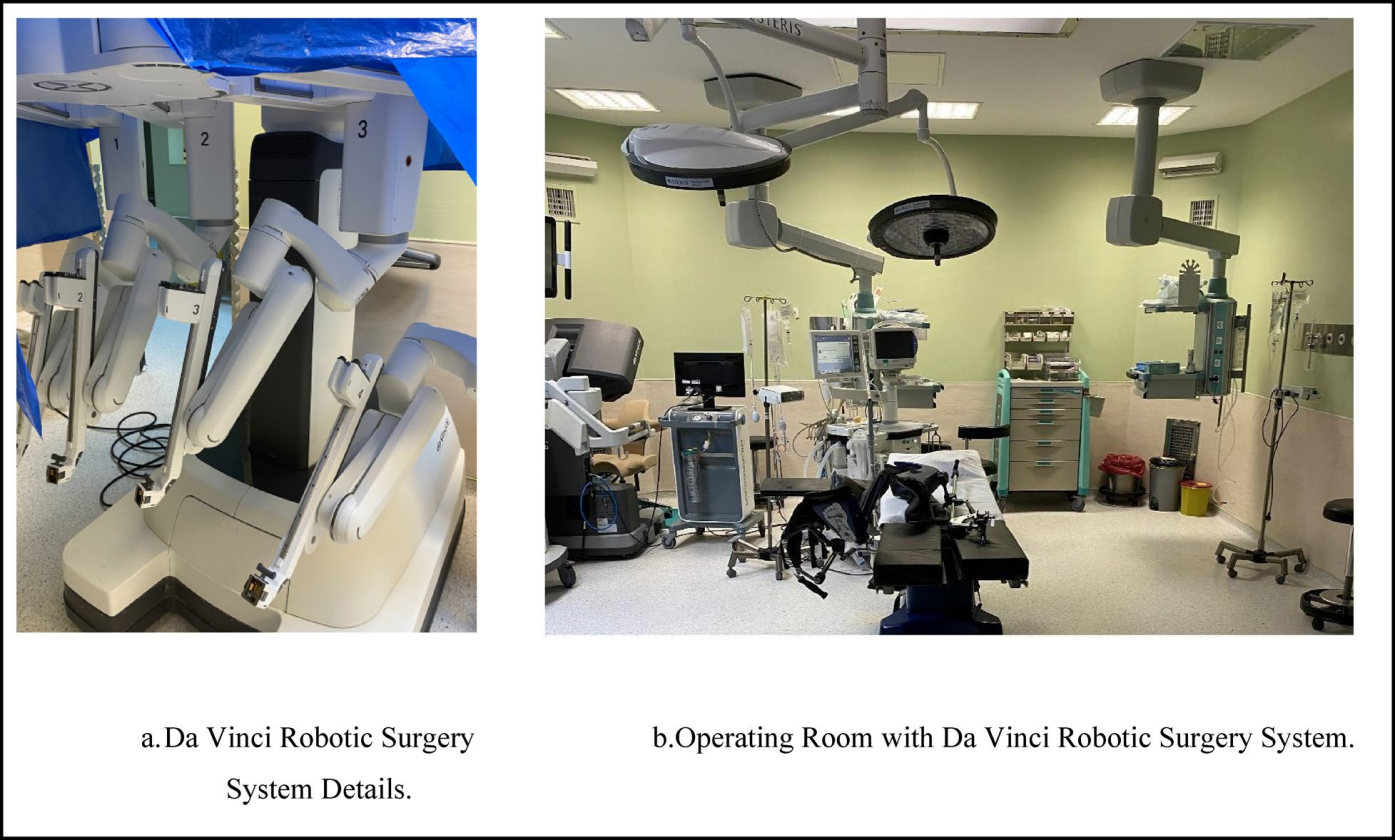
Da Vinci robotic surgery system. Photographs taken by co-author Faruk Yilmaz with permission in the research context hospital and published under a Creative Commons Attribution (CC BY) license.

Introduced in the mid-2000s, the Da Vinci Robotic Surgery System has been widely adopted in 40 hospitals across Turkey, particularly for cancers of the kidney, prostate, and bladder, and some gynecological surgical procedures. Nonetheless, the high operational costs of the Da Vinci Robotic Surgery System remain a significant barrier to its broader adoption. For this study, we selected a prominent and large public hospital known for its emphasis on education and research. This hospital was selected for its pioneering implementation of the Da Vinci Robotic Surgery System in 2010, employing the system since then across multiple specialties, such as urology, general surgery, cardiovascular surgery, and, to a lesser degree, otolaryngology (Fig. 1).

### Sample

While the sample size is modest, it aligns with established qualitative research standards that prioritize depth and richness of insight over breadth, particularly when exploring complex, context-dependent phenomena such as human-robot collaboration in surgical settings^35–37^. Due to the time- and labor-intensive nature of their profession, healthcare workers who use robotic systems are a challenging group to sample. To gather data effectively, we used a combination of purposeful sampling^38^ and snowball sampling, targeting healthcare professionals at various levels with differing experiences using healthcare robots. This approach enabled us to focus on participants who could provide in-depth insights and help identify emerging themes, rather than attempting to generalize findings across a broader population^36^. Previous studies on robotic surgery staff have often relied on interviews within a single specialty, limiting the generalizability of findings^39^.

For this study, we conducted eleven semi-structured interviews with varied and representative medical staff, including seven surgeons, three nurses, and one anesthesiologist, all of whom work closely with robotic systems in surgical and healthcare settings (Table 1). To ensure balanced representation, we included five female and six male participants, reflecting the gendered nature of healthcare regulations concerning work schedules and responsibilities^40^. Each interview, lasting between twenty and sixty minutes, took place face-to-face within the participants’ work facilities. All interviews were recorded, except for one participant who opted out of recording.

**Table 1:** Interviewee characteristics. *P: professor, A/P: associate professor; ** no other category reported.

No	Alias	Role and Title^*^	Sex^**^ (F/M)	Professional Experience with Surgery Robots (years)	Total Professional Experience (years)	Administrative Task	Interview	
							Date	Duration (minutes)
1	Can	Urology Surgeon	M	5–10	> 20	Administrative manager	10/13/2022	20
2	Kaan	Cardiovascular Surgeon, A/P	M	5–10	> 15	Education and administrative responsibility	10/13/2022	25
3	Fatma	Otolaryngology Surgeon, A/P	F	5–10	> 15	-	10/14/2022	20
4	Ali	Urology Surgeon, P	M	5–10	> 20	Education manager	10/21/2022	25
5	Sarp	General Surgeon, A/P	M	< 5	> 20	-	10/21/2022	40
6	Ada	Nurse	F	5–10	> 15	-	11/11/2022	50
7	Havva	Nurse	F	5–10	> 20	-	11/11/2022	30
8	Emel	Nurse	F	5–10	5–10	-	11/11/2022	50
9	Mesut	Urology Surgeon, Assistant	M	< 5	5–10	-	12/30/2022	50
10	Emin	Neurosurgeon, A/P	M	none, due to the managerial role	> 20	Deputy chief	1/6/2023	50
11	Ece	Anesthesiologist	F	5–10	> 20	-	12/30/2022	60

### Research design

The interviewing researchers employed several strategies, informed by the literature, for engaging healthcare experts. These strategies included identifying and recruiting target participants, scheduling and preparing for interviews, and building rapport with the professionals involved^41^. A semi-structured interview guide was developed and pilot-tested prior to data collection. As Berg and Lune^42^ note, predetermined questions are advantageous in qualitative research; however, the use of probing questions, along with the flexibility to explore tangents, can deepen the researcher-participant relationship and enhance the richness of data^43^. Consequently, the interviewer used probes as needed to elicit additional detail, allowing for a comprehensive exploration of participants’ experiences and fostering a stronger connection between researchers and participants.

The interview questions (see Table 2) covered topics such as the perceived strengths and advantages of robotic systems like the Da Vinci Robotic Surgery System, as well as their weaknesses and limitations. Additional inquiries focused on the impact of robots on work experiences, examples of both successful and unsuccessful interactions, challenges encountered in surgical settings, burdens imposed on healthcare providers by robotic systems, and participants’ views on the future of human-robot collaboration in healthcare. The open-ended questions were drafted, partly modeling previous work on investigating AI attitudes among robotic surgery teams^44^.

**Table 2:** Human-robot relationship aspects and the interview questions.

Focus	Questions
The bright side of the relationship: robots augment surgery	What are the strengths and opportunities of robots (i.e., the Da Vinci Robotic Surgery System) in healthcare processes? In which specific contexts do they work especially well?
	How have the possibilities provided by robots affected your work experience? Can you give an example of success?
The dark side of the relationship: robots’ risk/deteriorate surgery	What are the weaknesses and limitations of robots? In which specific contexts do you encounter problems? How have the limitations of robots affected your experience? Can you give an example of failure? Are there any situations where the robot restricts/hinders you in surgery/does not obey the orders, or causes delays? Did the robot make a mistake? What are the extra burdens (training, preparation, etc.) brought to healthcare providers by choosing robotic services?
Future projections of the relationship	What are your thoughts/feelings about humans and robots working together (regarding the level, form, design, and roles of the relationship)?
	To what extent do you think human-robot collaboration will reach in the future, and how do you think this will affect the future of healthcare?

### Data analysis

We adopted a systematic, iterative approach to data analysis that comprised two main stages: analysis and interpretation. First, the interviews were transcribed, translated, and returned to the original interviewers for verification, allowing the researchers to ensure that no essential details were lost or misinterpreted. After confirming the accuracy and context of the transcription and translation, we proceeded with the interpretive analysis.

To enhance the rigor of our qualitative analysis, two researchers independently conducted open, axial, and selective coding^38^. This parallel coding approach enabled us to surface divergent interpretations early in the process, which were then reconciled through collaborative discussion and iterative refinement. While formal calculation of inter-coder reliability (e.g., Cohen’s Kappa) was not employed due to the interpretivist orientation of the study, procedural reliability was upheld by cross-checking thematic codes and maintaining an audit trail of coding decisions (Miles, Huberman, & Saldaña^45^),. This strategy ensured analytical consistency and transparency while allowing for the emergence of nuanced insights from the data.

Our analysis followed three stages as outlined by Corbin and Strauss^38^: open coding, axial coding, and selective coding. In the open coding phase, we segmented the data and labeled recurring themes, concepts, and ideas. Two researchers conducted this manual coding independently, which allowed for a thorough examination of the data from multiple perspectives. During the axial coding phase, we organized the data into analytical categories, identifying three core themes through iterative sorting and refinement.

Selective coding focused on connecting these categories to broader narratives by revisiting and exploring their interrelationships. This stage involved summarizing the raw data, which enabled us to identify the primary themes related to robotic services literature. We then proceeded with data reconstruction, synthesizing the categorized data to uncover underlying stories and recognizing patterns within specific contexts. This final step allowed us to place themes within the broader healthcare and technological frameworks relevant to our study.

**Fig. 2:**
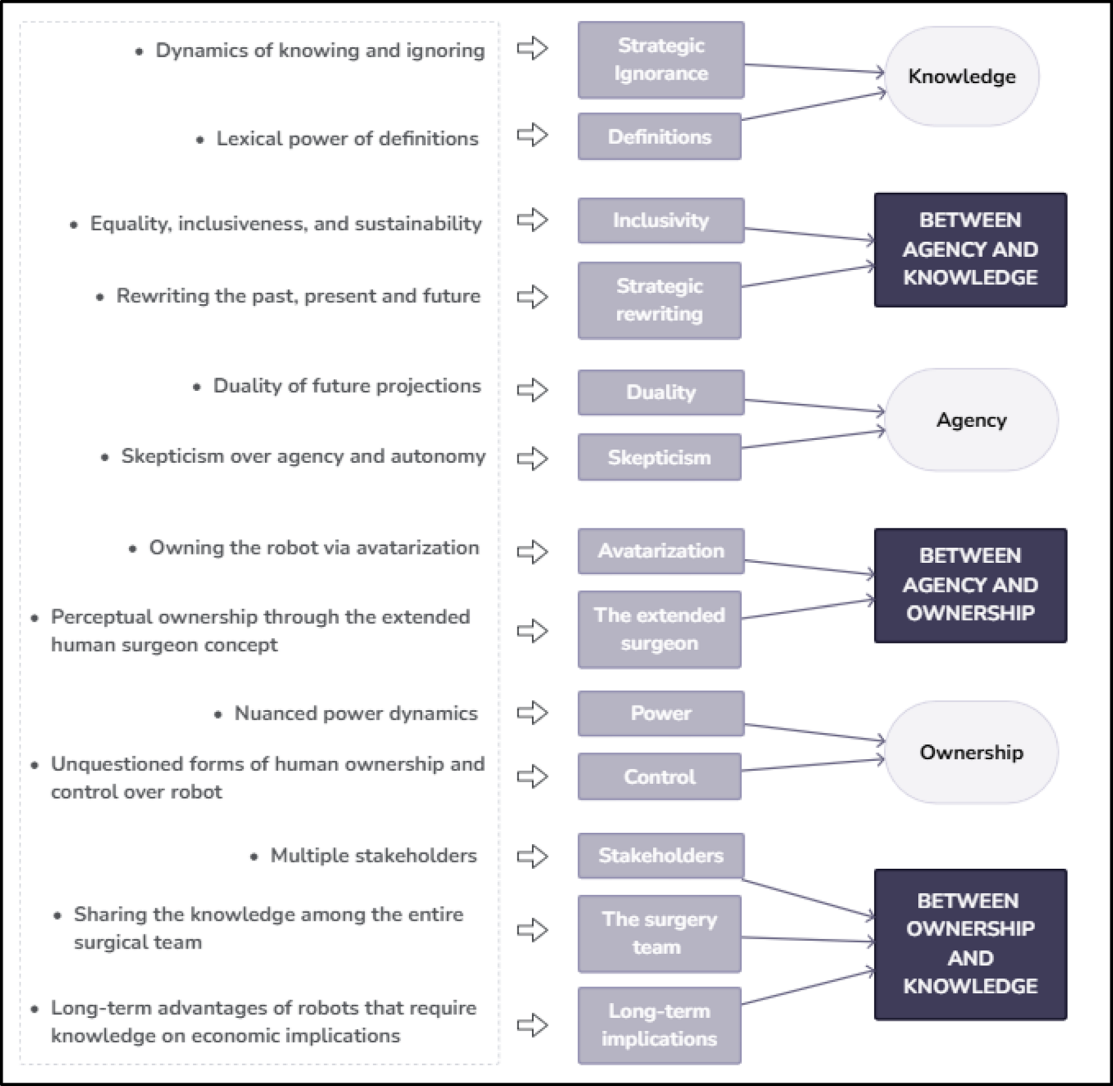
Data structure (authors’ own figure).

Subsequent meetings were held to refine and accurately label themes. These discussions facilitated comparisons of the most meaningful interview extracts, helping us to refine our coding framework and adjust our approach as necessary. This iterative process involved repeated theme comparisons and coding refinements (Fig. 2).

## Future research directions

Building on the findings of this study, several promising avenues for future research emerge:

1. Cultural and Gendered Dimensions of Human-Robot Collaboration.

Future studies can explore how cultural norms, values, and societal structures shape perceptions of knowledge, agency, and ownership in human-robot collaboration. For example, examining whether hierarchical versus egalitarian cultural contexts influence professional dynamics with robots can shed light on regional variations in acceptance and adaptation. Similarly, investigating how gendered expectations in traditionally male-dominated fields like surgery affect perceptions of robotic systems could offer critical insights into inclusivity and equitable access to training and roles.

2. Psychological and Professional Impacts of Avatarization.

As the concept of avatarization redefines the boundaries between humans and robots, research is needed to understand its long-term implications on professional identity, mental health, and job satisfaction. Does viewing robots as extensions of human expertise lead to greater professional pride, or does it blur accountability and increase pressure on individuals to perform flawlessly? Cross-sectoral studies in industries like logistics, education, or creative design, where robots are increasingly integrated into workflows, could offer comparative observations about how avatarization impacts professional dynamics and innovation.

3. Industry Comparisons in Automation Adaptation.

Comparative research across high-stakes environments, such as manufacturing, military operations, and space exploration, can identify commonalities and divergences in how humans adapt to automation. These studies could focus on the dynamics of team integration, power redistribution, and the balance of agency between humans and robots. For example, how do assembly-line workers perceive agency differently from healthcare professionals working with surgical robots? Understanding these differences could inform sector-specific policies and design principles for collaborative robotics.

4. Ethical and Policy Implications of Robotics in High-Stakes Environments.

Future research could examine more closely the ethical dilemmas and policy challenges posed by robotic systems in critical fields like healthcare and defense. How can regulations ensure that robotic adoption balances efficiency with equity, sustainability, and inclusivity? What frameworks can be developed to address the unintended consequences of automation, such as workforce displacement or the erosion of team cohesion?

5. Robotic Training Programs and Workforce Development.

Research can examine the effectiveness of current robotic training programs and propose more inclusive and adaptive training frameworks. For instance, how can training be designed to reduce the knowledge hierarchies observed in this study and empower all members of a surgical team, from surgeons to nurses? Further, exploring ways to make training more accessible in resource-constrained settings could address disparities in the adoption and use of robotic systems globally.

6. Robotics, Sustainability, and Resource Efficiency.

With concerns about the high costs and proprietary nature of robotic systems, future research can investigate how innovations in design and manufacturing could reduce costs and improve resource efficiency. Exploring circular economy principles, such as the reuse or recycling of robotic components, could offer solutions to the sustainability challenges identified in this study.

7. Human-Technology Co-Adaptation.

Finally, longitudinal studies can explore how humans and robots co-adapt over time. Do professional behaviors and attitudes toward robots evolve with prolonged exposure, or do initial resistances persist? This line of inquiry could offer suggestions about how organizations can better support the cultural and psychological adjustments required for successful human-robot collaboration.

Future research that addresses these questions can strengthen our theoretical knowledge about human-robot collaboration and inform practical strategies for designing equitable, sustainable, and inclusive robotic systems across industries.

## Data Availability

The datasets generated and/or analyzed during the current study are not publicly available due to confidentiality agreements and the sensitive nature of the questions asked. Interviewees were assured that raw data would remain confidential and would not be shared. However, de-identified data may be available from the corresponding author upon reasonable request.
